# The implementation of an artificial intelligence-based software for large vessel occlusion stroke improves door-to-puncture time at a comprehensive stroke center

**DOI:** 10.1007/s11739-025-04183-z

**Published:** 2025-11-20

**Authors:** Murtaza Akhter, Christine Ibrahim, Kara Nolan, Olivia Igoe, Christine Mae Anzardo, Christopher J. Love

**Affiliations:** 1 Penn State Hershey Medical Center, Hershey, PA USA; 2 HCA Healthcare, Miami, FL USA; 3 Viz.ai, San Francisco, CA USA

**Keywords:** Artificial intelligence, Large vessel occlusion, Thrombectomy, Stroke

## Abstract

Endovascular thrombectomy (EVT) is the standard treatment for large vessel occlusion (LVO) acute ischemic stroke (AIS), with a target door-to-device time of under 90 min. While prior reports have indicated that artificial intelligence (AI) software can reduce door-to-procedure times, we set out to study its impact in our own patient population. A retrospective cohort study was conducted at a comprehensive stroke center located in a densely populated, urban region consisting of a majority Hispanic population. Data were analyzed from 222 patients who underwent EVT between July 2018 and July 2022. The study compared workflow and patient outcomes before (*n* = 84) and after (*n* = 138) the implementation of an AI software (Viz LVO, Viz.ai, Inc.) in July 2020. The primary outcome was the fraction of EVT patients who received thrombectomy within 90 min. The proportion of patients undergoing thrombectomy within 90 min increased significantly from 26.5% (95% CI 17.9–36.8%) to 68.1% (95% CI 60.0–75.5%, *p* < 0.001). The median door-to-groin (DTG) time decreased from 109 min pre-Viz to 75 min post-Viz (*p* < 0.001). An interrupted time-series analysis confirmed a significant reduction in DTG time following the AI software implementation (*p* = 0.001). The percentage of AIS patients who received EVT went up between pre- and post-AI periods. No differences were found in TICI scores, length of stay, or mortality/hospice rates. Implementation of an AI-powered platform was associated with a significant increase in the fraction of patients who received EVT within 90 min and decrease in DTG time. An AI-based system helped optimize stroke care workflow in an urban center with a diverse patient population.

## Introduction

Acute ischemic stroke (AIS) is a debilitating disease, affecting 15 million people worldwide every year [1]. While treatment options were limited for years, there are now two effective therapies: tissue plasminogen activator and endovascular thrombectomy (EVT), which has become the standard of care for many patients, especially for those with large vessel occlusion (LVO) [2, 3]. However, since time is brain [4], these treatments are dependent on timely administration [5]; the American Heart Association has set a goal for door-to-device time within 90 min in 50% or more of eligible direct arrivals with acute ischemic stroke [6].

Our hospital, which treated close to 96,000 patients in 2023, is a comprehensive stroke center and Level 1 Trauma Center consisting of 424 beds and a predominantly Hispanic population (80–90% of patients identify themselves as Hispanic) [7]. We implemented an AI-based stroke software to try to improve stroke workflow. Prior reports have shown a post-implementation decrease of 12–22.5 min in door-to-groin (DTG) time [8–10]. We sought to investigate the effect of the implementation in our specific setting. The primary outcome was fraction of EVT patients who underwent thrombectomy within 90 min before vs after AI implementation. Exploratory outcomes included the pre–post-implementation median DTG times, percentage of AIS patients who received EVT, TICI scores, length of stay, and in-hospital outcome.

## Methods

The findings of this analysis are reported in accordance with the STROBE guidelines [11]. The study was approved by the Institutional Review Board of HCA Healthcare with a waiver of consent. Data in this report will be made available to any qualified investigator upon reasonable request of the corresponding author.

Our comprehensive stroke center collects data on its stroke patients for quality and certification purposes. We conducted a retrospective analysis of records for all non-transfer patients who presented to our emergency department between July 22, 2018 and July 24, 2022 and underwent EVT; Viz LVO was implemented in July of 2020. Viz LVO uses deep learning algorithms to analyze CTA images of the brain for LVOs in parallel to standard of care image interpretation. It also enables the previewing of available non-contrast computed tomography (NCCT) images and computed tomography perfusion (CTP) images as well as secure, HIPAA-compliant communication within the platform with other members of the stroke team. It is a notification-only, parallel workflow tool. Images that are previewed through the mobile application are compressed and not intended for diagnostic use beyond notification; however, the platform also allows for multiple physicians—including emergency, neurology, radiology, neurointervention, and anesthesiology—to communicate via HIPAA-compliant text messaging, phone calls, and telecommunication functionality.

To assess the effect of Viz LVO implementation, we compared median door-to-groin (DTG) time of EVT patients, fraction of EVT patients who underwent thrombectomy in under 90 min, median CTA to puncture time of EVT patients, percentage of AIS patients who received EVT, fraction with good TICI scores (defined as TICI 2b or higher), length of stay (LOS), and mortality and hospice rates in EVT patients before and after Viz LVO implementation. We also performed an interrupted time-series analysis to confirm whether time reductions corresponded to Viz implementation. The primary outcome was the fraction of EVT patients who underwent thrombectomy within 90 min; all other outcomes were exploratory. Our a priori power calculations confirmed that we had a sufficient sample size to test the primary outcome and DTG and CTA to puncture time exploratory outcomes for a two-sided type I error of 0.05 and 90% power. DTG time and CTA to puncture time were not normally distributed based on Shapiro–Wilk testing and visual inspection (Q–Q plot); differences were analyzed by Mann–Whitney U test, which was also used for National Institutes of Health Stroke scale (NIHSS) and LOS analyses given that these were not normally distributed. Chi-square tests were used for proportions and confidence intervals were reported using Mid-P exact. The time-series analysis was reported with regression coefficients and p values. Missing data were not imputed. Analyses were performed using R (version 4.4.2).

## Results

A total of 222 EVT patients were included in the analysis, of which 84 received care before the implementation of Viz LVO and 138 received care after implementation (Table 1). The most common occlusion site, which was extracted from the radiology report, was the M1 MCA (Table 2). The median DTG time was lower for patients who received care after implementation of the AI system (75 min vs. 109 min, *p* < 0.001). The fraction of patients who underwent thrombectomy in under 90 min improved significantly from 26.5% (95% CI 17.9–36.8%) pre-Viz to 68.1% post-Viz (95% CI 60.0–75.5%, *p* < 0.001).

**Table 1:** A summary of the key study results. Unless otherwise indicated, all analyses were conducted on the complete data set consisting of 84 pre-AI and 138 post-AI patients

	Pre-AI	95% CI or IQR	Post-AI	95% CI or IQR
Baseline characteristics	*N* = 84		*N* = 138	
% Female	51.4%	(43.1–59.7%)	58.3%	(47.6–68.5%)
NIHSS (median, IQR)^a^	15	(IQR 11–21)	16	(IQR 10–21)
Primary Outcome	*N* = 83		*N* = 138	
% < 90 min DTG	26.5%	(17.9–36.8%)	68.1%	(60.0–75.5%)
Exploratory outcomes				
DTG time (median min, IQR)^b^	109	(IQR 88–137)	75	(IQR 59–95)
CTA-read to puncture time (median min, IQR)^c^	63.5	(IQR 46–91)	45	(IQR 31.5–62)
TICI ≥ 2b^d^	93.5%	(86.2–97.6%)	90.3%	(84.4–94.5%)
Length of stay (median days, IQR)	12	(5–13)	10.4	(5–14)
Death or Hospice %	29.4%	(20.5–39.7%)	25.9%	(19.1–33.7%)

NIHSS, National Institutes of Health Stroke Scale; DTG, door-to-groin; and TICI, thrombolysis in cerebral infarction

^a^Pre-AI *N* = 81

^b^Pre-AI *N* = 83

^c^Pre-AI *N* = 76, Post-AI *N* = 136

^d^Pre-AI *N* = 77, Post-AI *N* = 134

**Table 2:** The distribution of occlusion site locations in patients undergoing endovascular thrombectomy in the pre- and post-AI periods. Note that if a patient had an occlusion in multiple locations, each location was counted (and therefore summating the numbers from each location yields a higher number than the total number of patients)

Occlusion location Pre-AI (84)		Post-AI (138)
Vertebrobasilar	8 (9.52% [CI 4.52–17.3%])	9 (6.52% [CI 3.23–11.6%])
PCA	3 (3.57% [CI 0.92–9.41%])	3 (2.17% [CI 0.56–5.80%])
ICA	15 (17.9% [CI 10.8–27.2%])	37 (26.8% [CI 19.9–34.7%])
MCA	68 (81.0% [CI 71.2–88.3%])	112 (81.2% [CI 74.0–87.0%])
ACA	1 (1.19% [CI 0.06–5.73%])	1 (0.72% [CI 0.04–3.52%])

In the included pre-Viz period, our center saw 739 AIS patients. In the included post-Viz period, we saw 763 AIS patients. Thus, the percentage of AIS patients who received EVT went from 11.2% (95% CI 9.10–13.7%) in the pre-Viz period to 18.1% (95% CI 15.5–20.9%) in the post-Viz period (*p* < 0.001).

**Fig. 1:**
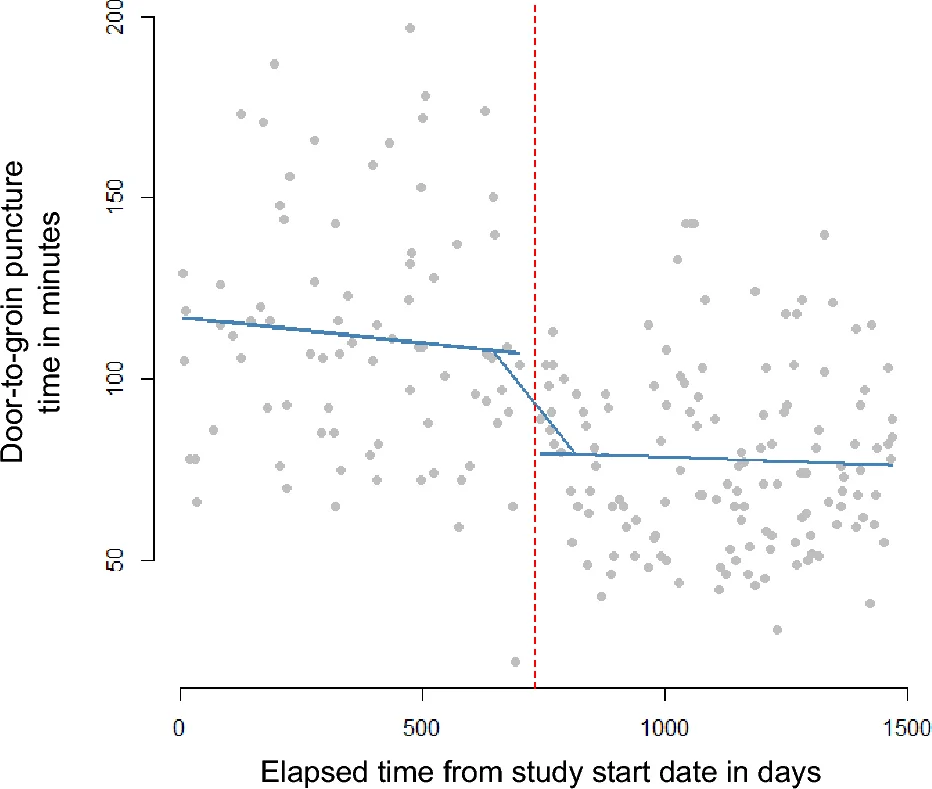
An interrupted time-series analysis of the door-to-groin puncture time (y-axis) versus elapsed time from study start date (x-axis) with the dashed red line indicating implementation of the AI-based system. There was no significant trend *during* the time period before (*p* = 0.37) or *during* the time period after (*p* = 0.63) the implementation; however, the AI system implementation was associated with a significant decrease in door-to-groin time (*p* = 0.001). A total of 221 patients were analyzed with 1 missing Pre-AI groin puncture time

An interrupted time-series analysis showed no significant trend *during* the time period before (*p* = 0.37) or *during* the time period after (*p* = 0.63) Viz implementation. However, the “interruption” of the Viz implementation per se led to a decrease in DTG time (*p* = 0.001; Fig. 1).

Similar percentages of patients underwent successful reperfusion (TICI score 2b or higher) in pre-Viz and post-Viz periods (93.5% [95% CI 86.2–97.6%)] and 90.3% [95% CI 84.4–94.5%] respectively). The median LOS for patients prior to AI implementation was 12 days (IQR 5–13 days) and after implementation was 10.4 days (IQR 5–14 days). Prior to AI implementation, 29.4% of patients (95% CI 20.5–39.7%) died or went to hospice, whereas after AI, 25.9% (95% CI 19.1–33.7%) died or went to hospice.

## Discussion

Our study showed that the implementation of an AI-powered decision support system yielded significant reductions in DTG times. There was no effect on LOS, successful reperfusion rates, or in-hospital mortality and/or discharge-to-hospice, but our study was not powered for these analyses. Additionally, the 90-day patient outcomes were unknown—a limitation of our retrospective study. However, it has been shown that in LVO stroke, time from stroke onset to reperfusion is one of the strongest determinants of outcomes [12, 13].

Our findings are in line with those of other groups. With respect to DTG time, Figurelle et al. showed similar decreases [14]. Other groups have also shown decreases in DTG time after AI software implementation that have tended to be smaller—from 12 to 22.5 min [8–10]—with the exception of Hassan et al., who showed an 87-min improvement [15]. Both the study by Hassan et al. and our study were based on a predominantly Hispanic population; however, their group’s patient population was predominantly of Mexican descent, whereas ours was predominantly of Caribbean and South American descent [16]. In a large registry analysis, DTG was shown to be delayed in Hispanic compared to non-Hispanic White patients in sites serving a minority Hispanic population [17]. Future work should investigate the potential for an AI-based system to improve workflow disparities. Possible improvements pre-imaging could come from new features that address patient-provider communication barriers and post-imaging from the expanded access to more rapid review of rural spoke imaging within the entire spoke-and-hub network on a single care coordination platform.

There could be multiple reasons for the observed decrease in DTG time. Our CTA-read to skin-puncture time was significantly lower after the AI software implementation and accounted for about half of the total DTG time difference. It is possible that additional time savings came from the ‘code stroke’ notification on the AI system—in particular, its speeding up the overall flow of stroke care by simultaneously notifying all relevant teams about a potential stroke. The downstream impact could be more expeditious transport to CT, scanner preparation by technicians, and access to imaging by treating neurologists and radiologists who are prepared for a potential stroke treatment. Thus, not only can the LVO be diagnosed more quickly, but the treatment can be pursued more rapidly (e.g., intervention suite is made ready, interventionist is available sooner, etc.), since all findings and communications are visible in real time across all relevant teams in a single application. Indeed, a multi-pronged approach for notifying people is needed to improve times with a complex process like stroke care [18].

While the observational nature of the study cannot exclude the contribution to the findings of other potential confounding factors, there were no major specific interventions or process improvements (besides the AI software implementation) that otherwise occurred within this period that would have necessarily affected DTG time. Furthermore, our time-series analysis showed a significant improvement of DTG time right after implementation of the AI-based system. This improvement occurred despite no significant trend toward improvement prior to the AI intervention; furthermore, the improvement persisted (as shown by no significant trend after the intervention on the time-series analysis).

Additionally, a significantly higher proportion of AIS patients received EVT in the post-implementation period. While we cannot conclude if this observation resulted from more eligible patients due to process improvements (quicker access to images and reads as well as faster communication) or national trends in the increased use of EVT, it should be noted that one study reported a relative flattening of the annual growth curve for EVT procedures in the U.S. from 2018 to 2021 [19], which encompasses most of the period of the present analysis. Furthermore, national trends would not explain why our site showed no trend in DTG time prior to or after the AI intervention but showed a significant and persistent drop immediately after the intervention.

### Limitations

There are multiple limitations to this study. First, as a single-center analysis, the findings may not be generalizable. Indeed, few hospitals in the country have as high of a proportion of its patients identifying as Hispanic and virtually none with the specific Latin American population (i.e., large proportion with Cuban descent) as ours. However, our findings are in line with those from other groups and confirm a prior single-center result involving a predominantly Hispanic population. Furthermore, our study was performed at a comprehensive stroke center and excluded transfer patients; AI decision support may have a different effect at hospitals that are not already comprehensive stroke centers. Given the retrospective nature of the study, we cannot exclude other confounding factors such as workflow efficiency gains over time associated with the increased use of and experience with EVT procedures—especially with expanded indications in 2018 (study period start date) and continued publication of positive EVT trial results over time. However, our interrupted time-series analysis, which accounts for pre-intervention trends, showed a clear drop-off in door-to-groin times right after implementation of the AI software, with no significant decreases in times occurring before (or after) this point. Another limitation is the lack of 90-day functional assessment or neurological outcomes data, which would require a very large sample size with strict inclusion criteria [8].

## Conclusion

Implementation of an AI-powered platform improved multiple AIS workflow metrics and significantly increased the fraction of patients who received EVT within 90 min. These findings suggest that AI-augmented care can optimize stroke management in a high-volume, diverse urban center.

## Data Availability

Data in this report will be made available to any qualified investigator upon reasonable request of the corresponding author.
